# Distinct ventral stream and prefrontal cortex representational dynamics during sustained conscious visual perception

**DOI:** 10.1016/j.celrep.2023.112752

**Published:** 2023-07-07

**Authors:** Gal Vishne, Edden M. Gerber, Robert T. Knight, Leon Y. Deouell

**Affiliations:** 1 Edmond and Lily Safra Center for Brain Sciences, The Hebrew University of Jerusalem, Jerusalem 9190401, Israel; 2 Helen Wills Neuroscience Institute, University of California, Berkeley, Berkeley, CA 94720, USA; 3 Department of Psychology, University of California, Berkeley, Berkeley, CA 94720, USA; 4 Department of Psychology, The Hebrew University of Jerusalem, Jerusalem 9190501, Israel; 5 Twitter: @neuro_gal; 6 Twitter: @deouell; 7 Lead contact

## Abstract

Instances of sustained stationary sensory input are ubiquitous. However, previous work focused almost exclusively on transient onset responses. This presents a critical challenge for neural theories of consciousness, which should account for the full temporal extent of experience. To address this question, we use intracranial recordings from ten human patients with epilepsy to view diverse images of multiple durations. We reveal that, in sensory regions, despite dramatic changes in activation magnitude, the distributed representation of categories and exemplars remains sustained and stable. In contrast, in frontoparietal regions, we find transient content representation at stimulus onset. Our results highlight the connection between the anatomical and temporal correlates of experience. To the extent perception is sustained, it may rely on sensory representations and to the extent perception is discrete, centered on perceptual updating, it may rely on frontoparietal representations.

## In brief

How is ongoing visual experience represented neurally? Vishne et al. decode images lasting different durations from intracranial electrophysiology, uncovering distinct representation dynamics across the human brain: sustained and stable in occipitotemporal cortex and transient in frontoparietal areas. This sheds light on the spatiotemporal correlates of experience encoding by the brain.

## Graphical Abstract

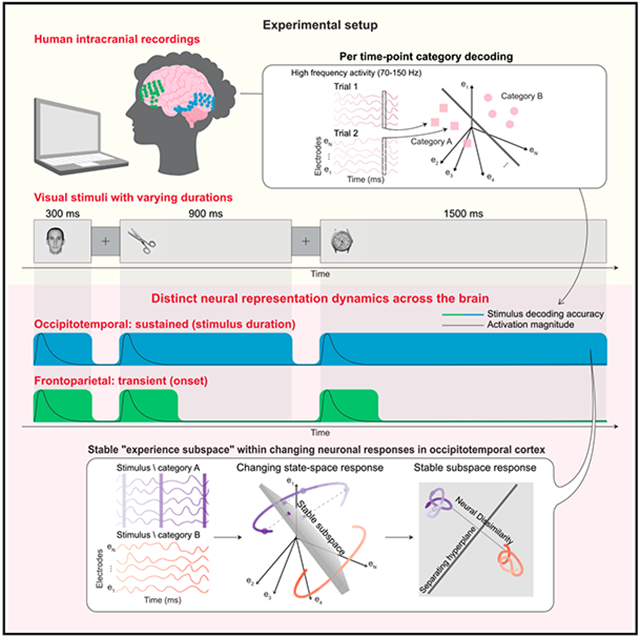

## INTRODUCTION

In essence, every perception has non-zero duration–we gaze at a tree for some time, then shift our gaze to look at a fly that just landed on the table only to take off after a few seconds. All these experiences have a content (a tree, a fly) that extends not only in space but also in time. Most discussions of the neural correlates of consciousness (NCC), defined as the minimal set of mechanisms that are together necessary and sufficient for any one specific experience,^1^ addressed the anatomical location in the brain that gives rise to the experience, while time has received considerably less attention in the NCC literature. Introspectively, it seems that our experience unfolds continuously, in parallel with the sequence of events; thus, we would expect that our experience of gazing at the tree was longer than the quick glance at the fly. However, this intuition is complicated by the existence of postdictive effects, when a current stimulus influences the experience of prior events.^2,3^ To account for this, some have argued that we are not continuously conscious, but rather we are conscious at discrete moments in time.^4,5^ The anatomical component of the NCC is also debated. One major point of contention involves the role of the prefrontal cortex compared with high-level sensory cortices.^6,7^ Multiple previous studies have found prefrontal responses to be associated with stimulus awareness, yet more recently, it was argued that this is a byproduct of the reporting procedure and not a signal pertaining to awareness per se.^8^

To date, searches for the anatomy of the NCC (where) and the temporal progression (when) of consciousness have progressed largely in parallel, as most studies have focused on the transient onset or change-related responses without examining the ubiquitous periods of stationarity between the changes.^9-11^ This is a critical challenge, as theories linking conscious experience to the brain should be able to account for experience of sustained events, not only that of stimulus onsets or changes. Importantly, the few studies that examined the full temporal dynamics of responses to longer stimuli found that activity in high-order visual regions drops dramatically shortly after the initial onset response, independent of stimulus duration.^12,13^ However, these studies focused on activation dynamics and did not examine content representation over time, which is the focus of the current study. Additionally, they focused exclusively on visual regions without addressing activation or representation in the frontoparietal cortex. Testable predictions regarding these issues were recently put forward in an adversarial collaboration aiming to adjudicate between two theories,^14^ global neuronal workspace theory (GNWT)^15^ and integrated information theory (IIT).^16^

**Figure 1.**
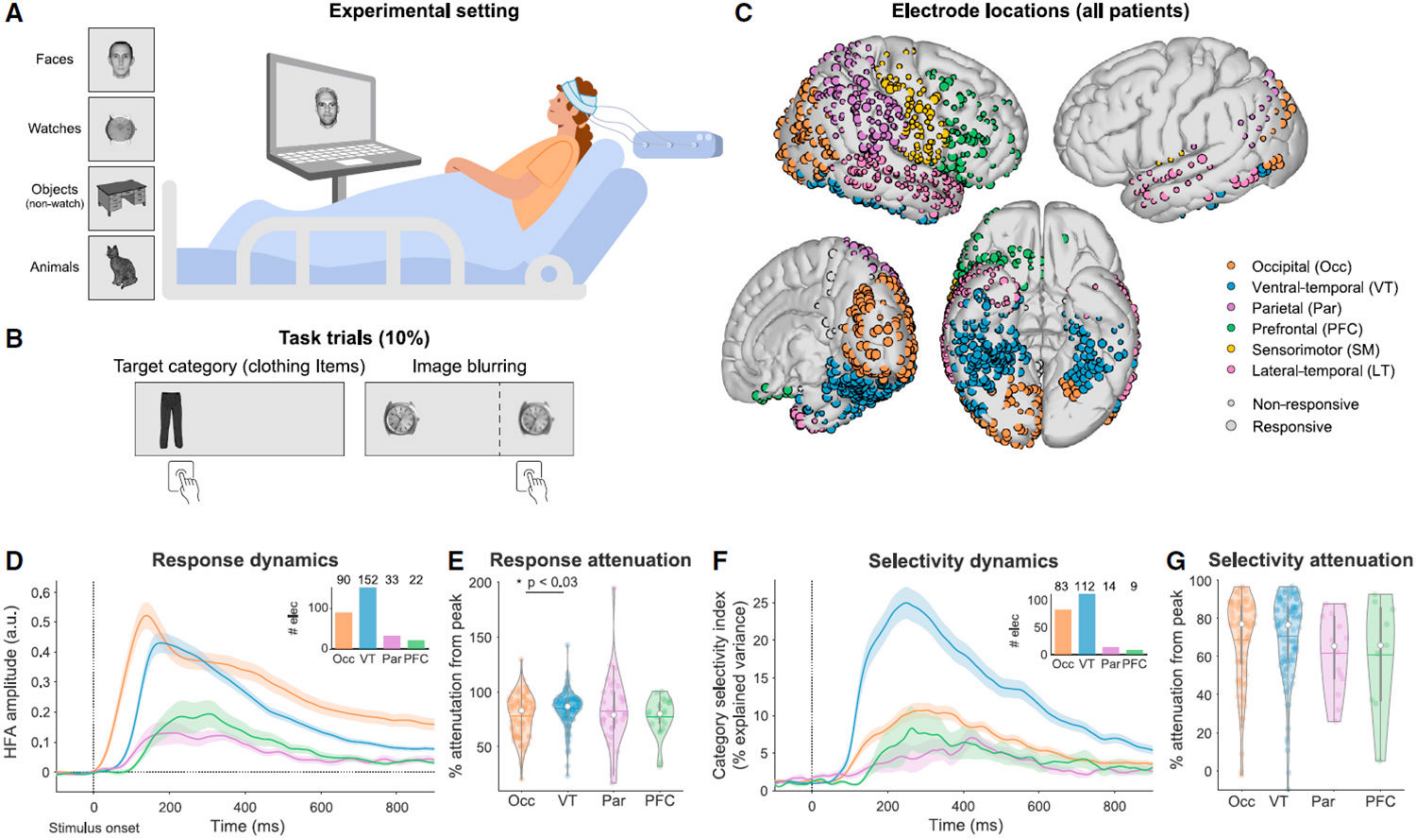
Experimental setup and design, electrode locations, and single-electrode response dynamics showing substantial attenuation after the onset response (A) Experimental setup and example images from the four categories. (B) Two target types (together 10% of trials): (1) clothing items and (2) blurring of the image. (C) Electrode locations (pooled across patients), colored by ROI. The same color scheme applies for all figures. See Tables S1C and S1D. (D) HFA response dynamics relative to the prestimulus baseline (positively responding electrodes). To highlight response dynamics, for each electrode, only trials from categories it was responsive to were included. Shaded area: SEM across electrodes (number shown in the inset). See also Figures S1A-S1E. (E) Relative attenuation in HFA responses from peak to 800–900 ms (peak minus end activity relative to peak; attenuation >100% when end activity is lower than baseline levels). Colored dots: single electrodes (same as in D), colored horizontal lines: mean across electrodes, white dots: median across electrodes, gray vertical bars: interquartile range, contour lines: kernel probability density estimate. Black horizontal lines and asterisks: significant post hoc differences between ROIs (Tukey-Kramer method). (F) Category selectivity dynamics in category-selective electrodes (η^2^ expressed as percentage of explained variance from a one-way ANOVA between categories). Notations as in (D). See Figures S1F-S1H for electrode locations and single-electrode properties. (G) Relative attenuation in selectivity, higher numbers indicate stronger attenuation. Notations as in (E). (D–G) Image durations ≥900 ms.

Here, we address these fundamental questions by examining the spatiotemporal neural representation of clearly visible images of different durations (300–1,500 ms) in ten human patients with drug-resistant epilepsy implanted with subdural electrodes for clinical purposes (Figure 1A; Tables S1A-S1C). To maintain attention, responses were required for 10% of the trials that were not analyzed, excluding any report-related signals (Figure 1B). Using multiple presentation durations allows us to distinguish between responses to the onset of a stimulus and signals tracking the ongoing stimulus presence. Presenting a diverse set of images enables us to identify signals tracking the content of experience, not only whether an image was shown or not (which was the focus of our previous work using this dataset^12^). We use this to examine visual representation over time at the level of visual categories (e.g., faces/objects) and at the level of single exemplars (e.g., specific objects). Within the object category, we analyzed separately a subcategory of watches, which bear low-level visual similarity to faces.^17,18^ To foreshadow our results, we find that despite considerable variability in moment-to-moment activation levels, the distributed population representation of visual content (category and exemplar level) in sensory regions is stable, paralleling the stimulus presentation. Further, we find transient visual representation in the prefrontal cortex, despite the lack of overt report. These findings confirm predictions of both theories delineated in the recent adversarial collaboration.^14^ More broadly, these results highlight the importance of addressing the NCC within a temporal context: to the extent conscious experience is continuous, it may rely on sensory representations, and to the extent experience is discrete, it may rely on prefrontal representations. Thus, by studying visual experience and representation beyond the onset, we reveal a connection between spatial (anatomical) and temporal understanding of consciousness.

## RESULTS

We measured broadband high-frequency activity (HFA; 70–150 Hz; STAR Methods), shown to reliably track local neuronal activity,^19,20^ in six *a priori* regions of interest (ROIs) defined anatomically (Figure 1C; Table S1D). Of 907 noise-free electrodes, we focus on 430 that were visually responsive, defined as significant HFA modulation relative to a 200 ms prestimulus baseline, for at least one category in at least one of four non-overlapping 200 ms time windows between 100 and 900 ms after stimulus onset (considering only images presented for 900 ms or longer; see STAR Methods and Table S1E for more details). Thus, electrode selection did not involve any category or temporal selectivity. Analyses focus on the four visually responsive regions: occipital (Occ), ventral temporal (VT), parietal (Par), and prefrontal cortex (PFC); other regions are shown in the supplemental information. With the exception of Figures 1D and 1E, all analyses in the main text include both positively (increased activity, >80% of sites) and negatively responding (decreased activity) electrodes. Similar results were obtained using positive electrodes only (supplemental information). To increase the number of trials in each category, we pool all images presented for 900–1,500 ms for all analyses except for Figure 4, where we consider each stimulus duration separately.

### Response magnitude and category selectivity in single electrodes is substantially attenuated after the initial onset response

We first examined the single-electrode HFA response dynamics to images presented for 900 ms or longer (Figure 1D). Response magnitude was higher in Occ and VT relative to the PFC and Par (one-way ANOVA, F(3,293) = 12.5, p < 10^−6^; Figure S1A) and peaked earlier in Occ relative to all other regions (F(3,293) = 15.73, p < 10^−8^; Figure S1B; see Figures S1C-S1E for response dynamics in other regions). Importantly, despite the continued presentation of the stimulus, response magnitude in all ROIs was substantially attenuated after the onset response, with ~5-fold reduction in activity by 800–900 ms after onset relative to peak response (mean attenuation ± SEM of electrodes from all four ROIs: 81.8% ± 1.1%). Attenuation magnitude slightly varied between regions (one-way ANOVA, F(3,293) = 2.98, p < 0.033; Figure 1E), with post hoc tests revealing only larger attenuation in VT compared with Occ (Tukey-Kramer method, p < 0.03, Cohen’s d = 0.42). VT and Occ showed strong responses at the onset, and even after the prominent response attenuation, their responses stayed (on average) higher than baseline levels (see our prior analysis of this data in Gerber et al.^12^ for more detailed discussion of this point).

Of 430 responsive electrodes 236 showed significant category selectivity in at least one 200 ms time window (one-way ANOVA between categories; STAR Methods; Figure S1F). In line with the general reduction in the magnitude of responses, by 800–900 ms, category selectivity (η^2^ values from temporally resolved ANOVA) declined by an average (±SEM) of 77.4% ± 1.1% relative to peak selectivity (Figures 1F-1G, S1G, and S1H; no significant differences between regions, F(3,214) = 1.09, p > 0.3). Thus, both response amplitude and category selectivity in single electrodes were substantially attenuated after the onset response, despite the continued presence of the stimulus.

### Multivariate state-space dynamics in sensory regions track the duration of the stimulus

**Figure 2.**
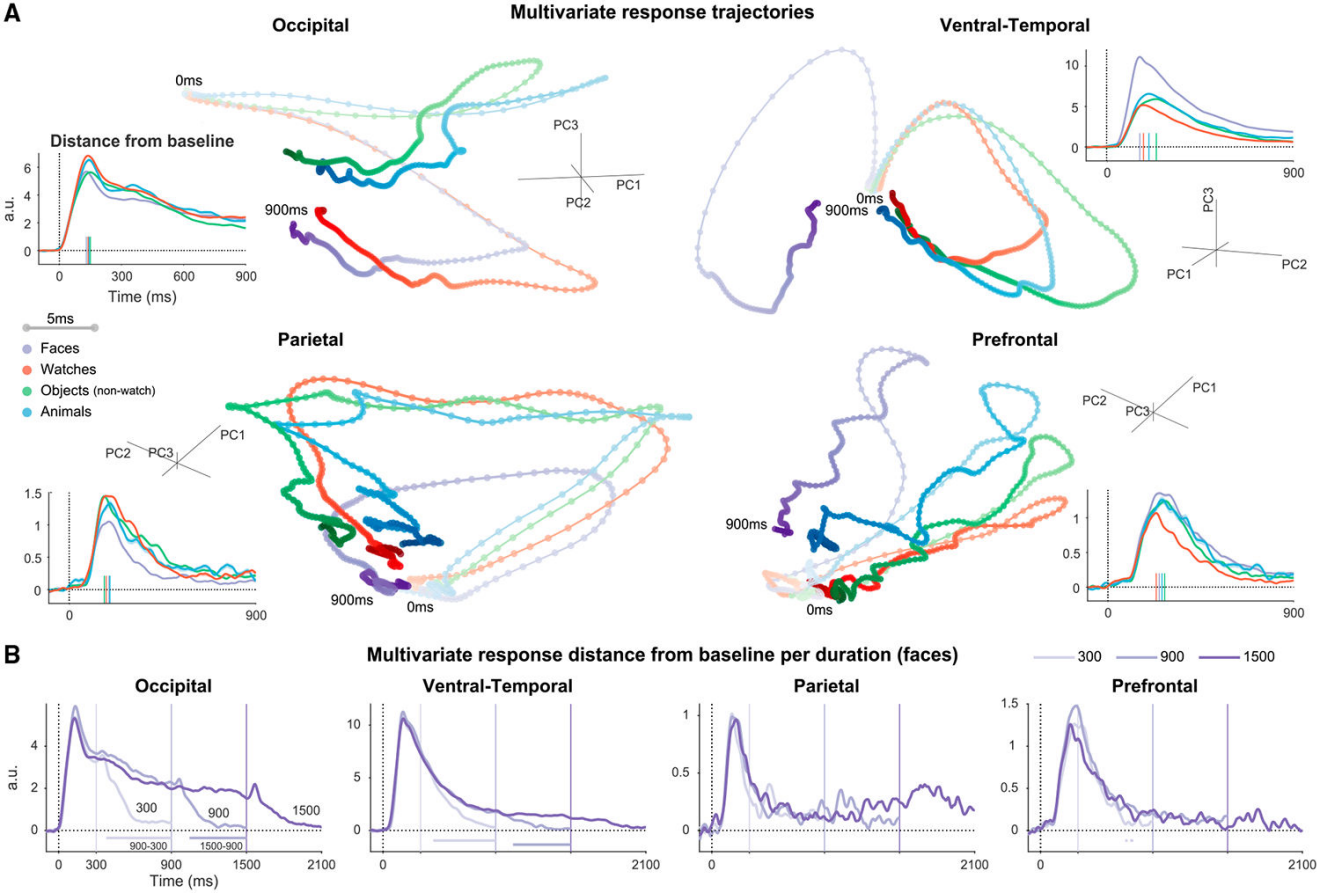
Multivariate state-space dynamics in sensory regions track the duration of the stimulus (A) State-space trajectories per category (image durations ≥ 900 ms; first 3 principal components using all responsive electrodes, responses in each category averaged prior to principal-component analysis [PCA]; PCA was performed solely for visualization purposes). Trajectory lines are darker and thicker as time progress, dots are 5 ms apart. Insets: point-by-point distance of each trajectory from the (baseline) prestimulus state (computed using the full response, prior to PCA); colored vertical lines on the abscissa: peak distance times. See Figures S2 and S3E for extended analysis of state-space trajectories. (B) Dynamics of distance from baseline (face images, see also Figure S3A; for other categories, see Figure S3B; baseline subtracted for presentation purposes). Offsets marked by vertical lines with corresponding hues. Horizontal bars: time points of significant differences between durations (max-statistic permutations, p < 0.05; 1,500 vs. 900 ms, 900 vs. 300 ms; colors correspond to the shorter duration in the contrast). Traces are cropped 600 ms after stimulus offset (shortest inter-stimulus interval, ISI). Absolute distances are comparable within regions at different time points, not between regions, as magnitude is dependent on the number of electrodes. See also Figures S3C and S3D.

To understand how information is encoded in patterns of distributed activity, we examined the multivariate state-space trajectory in response to each category separately^21-23^ (Figures 2A and S2A; all images presented for 900 ms or longer). We quantified multivariate activation using the point-by-point distance of the neural trajectory from the baseline state (prestimulus state, when no stimulus was presented; insets). Similarly to the single-electrode activation profiles, the multivariate activation increased rapidly after the onset, followed by marked attenuation (reduction of ~80% from peak response to 800–900 ms in all regions except Occ; Figure S2B). The decay of activity was substantially slower than the rise of the onset response, especially in Occ and VT regions (see Figures S2C-S2D for analysis of state transition speeds). Separating the responses to stimuli of different durations (faces: Figures 2B and S3A; watches: Figure S3B) shows that despite the amplitude attenuation after the onset, multivariate responses in VT and Occ precisely tracked stimulus presence, with significant differences comparing 900 with 300 and 1,500 with 900 ms stimuli, emerging shortly after the offset of the shorter stimulus (permutation test with max-statistic control for multiple comparisons, p < 0.05). Responses in the PFC and Par did not show this profile of duration dependence. Similar results were obtained by comparing trajectories directly (without considering the distance to the prestimulus state; Figures S3C and S3D).

### Visual category representation is sustained and stable in sensory regions and transient in frontoparietal regions

**Figure 3.**
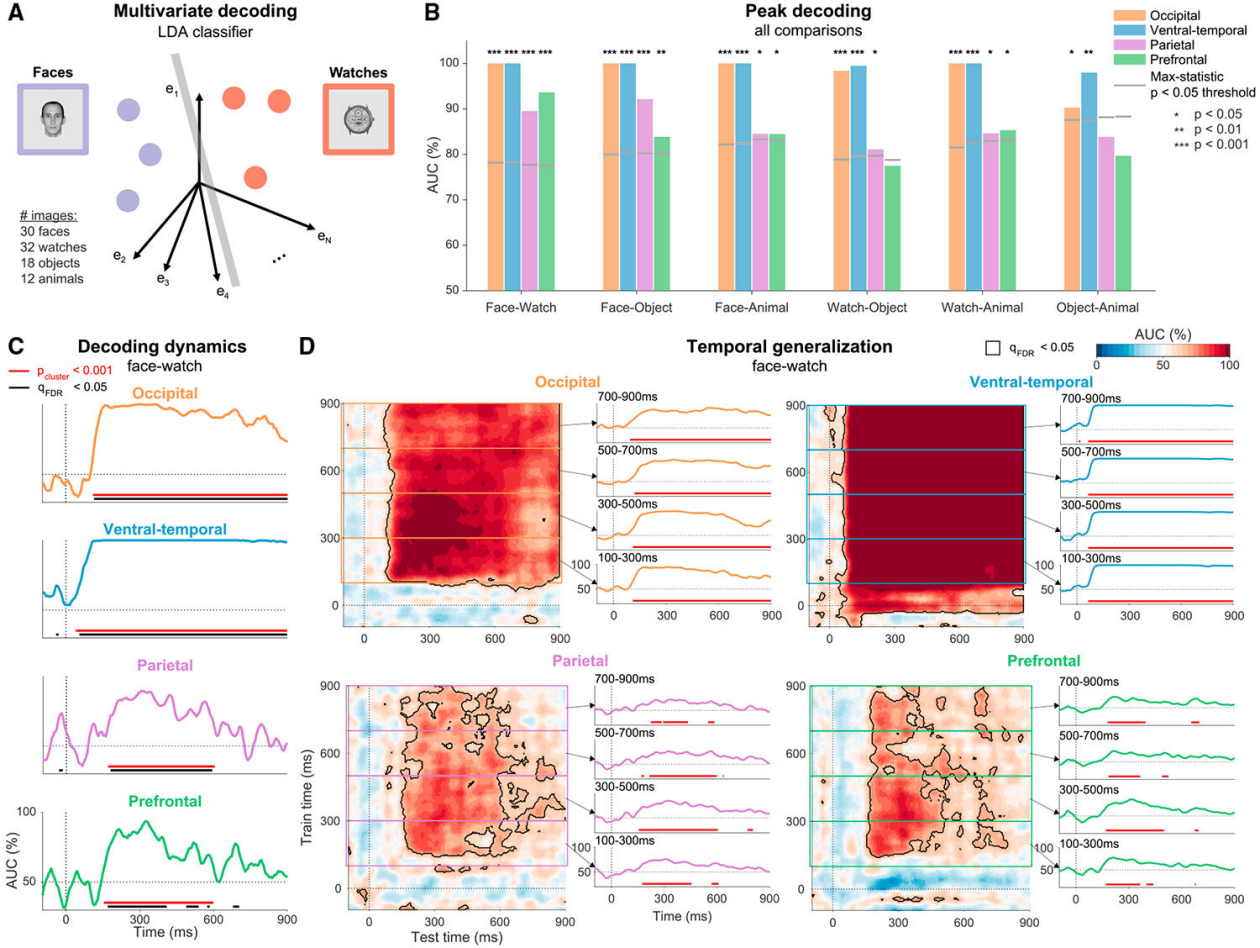
Visual category representation is sustained and stable in sensory regions and transient in frontoparietal regions (A) Schematic illustration of decoding for a single time point: colored dots represent single-trial responses; a gray bar represents the linear classifier. (B) Peak decoding. Significance computed by permutation testing. Gray horizontal lines: significance threshold (max-statistic permutation testing; threshold is higher for comparisons involving categories with less exemplars). (C) Decoding dynamics (face-watch; other comparisons and direct comparisons between regions: Figure S4). Dashed lines: stimulus onset and chance level. Red bars: significant clusters by cluster-based permutations, black bars: significant points by point-by-point permutation testing (FDR corrected). (D) Temporal generalization matrices (face-watch; other comparisons are shown for VT and Occ in Figures S5D and S5E). The diagonal (training and testing on the same time point) corresponds to the time courses in (C). Black contour: contiguous points significant by point-by-point permutation testing (FDR corrected). Right-side plots: mean generalization dynamics for 200 ms blocks of training time. Red bars: testing points significant for ≥50% of training points in the range. (B–D) All stimuli durations ≥900 ms. See Figures S5 and S6 for single-patient results, analysis of Occ and PFC subregions, and control analyses ruling out the contribution of ocular muscle activity to PFC decoding.

Next, we examine the representational content of the multivariate responses using time-resolved single-trial decoding^24,25^ on a subset of 92 unique images that were shown to all patients for 900 ms or longer. Similar conclusions were obtained by examining the dispersion between state-space trajectories (Figure S3E). We trained linear classifiers for each ROI to distinguish between each pair of categories, using the HFA responses across electrodes as features (Figure 3A). Classifier performance was evaluated using the area under the curve (AUC) of the receiver operating characteristic curve (ROC). Peak decoding was significantly higher than chance in both occipitotemporal and frontoparietal regions (Figure 3B; mean ± SEM across comparisons: VT, 99.6% ± 0.3%; Occ, 98.1% ± 1.6%; PFC, 84.1% ± 2.3%; Par, 86% ± 1.7%; one-sided max-statistic permutation test, VT and Occ all p_perm_ < 0.002, except Occ_object-animal_ p_perm_ < 0.022, PFC and Par all p_perm_ < 0.039, except object-animal in both regions and PFC_watch-object_). Thus, category information was not limited to traditional visual areas.

However, the temporal profiles of decoding performance were dissimilar across regions (Figure 3C focuses on the face-watch comparison, and all comparisons are shown in Figure S4A; direct time-resolved contrasts between regions are shown in Figure S4B). In VT and Occ, significant clusters^26^ emerged early after stimulus onset and persisted throughout stimulus presentation (all p_cluster_ < 0.001, red horizontal bars in Figure 3C; confirmed also with false discovery rate [FDR]-corrected point-by-point permutations, black horizontal bars). Despite the substantial attenuation in response magnitude and in single-electrode selectivity in both regions (Figure 1), category decodability decreased only minimally throughout this time (face-watch mean AUC 100–900 ms: VT, 99.8%; Occ, 92.4%). These results did not stem from a single patient; high and persistent decoding performance was observed in the majority of patients with electrodes in these regions (Figures S5A and S5B). Similar results were found for both retinotopic and non-retinotopic regions of Occ (identified using a probabilistic map of visual topographic areas^27^; Figure S5C).

In contrast to the visual areas, significant category decoding in the PFC and Par was transient and mostly limited to ~150–600 ms after stimulus onset (face-watch, both p_cluster_ < 0.001; see Figures S6A and S6B for single patients). Onset times were delayed relative to the appearance of category information in sensory regions, consistent with the idea that content-selective activity in frontoparietal regions only emerges after activity in sensory areas reaches a critical level.^15^ We repeated the analysis separately for the orbitofrontal cortex (OFC) and lateral prefrontal cortex (LPFC), as these parts of the cortex belong to partially distinct networks considering cytoarchitectonics, connectivity patterns, and function.^28,29^ Category information was significantly decodable from both subregions, though it was more prominent in the OFC relative to the LPFC (Figure S6C). Given the intense debate about the role of prefrontal representation in conscious awareness,^15,30^ we applied several controls that ruled out the contribution of ocular muscle artifacts to PFC decoding (STAR Methods; Figures S6D-S6E; as the LPFC is less susceptible to ocular-muscle artifacts, these analyses focus on OFC electrodes). Coverage in these regions was less comprehensive than in sensory regions (Figure 1C), which raises the possibility that the transient nature of category information in these regions stems from the reduced coverage. However, increasing the number of PFC electrodes by not limiting analysis to responsive electrodes led to similar results (Figure S4A). We also repeated the analysis in Occ and VT after reducing the number of electrodes to match coverage in the PFC, which nonetheless resulted in sustained category information in these regions (Figure S4C). Thus, the PFC transiently represents category information even though no overt report was required for any of the stimuli used in the analysis.^8,31^

These findings show that visual areas provide reliable category information for as long as the image is presented, not only at the onset. This sustained decoding could stem from a series of changing discriminating patterns or a single sustained state. To address this, we applied the temporal generalization method,^32^ in which classifiers are trained on data from each time point separately, but each classifier is tested on all time points, resulting in a temporal generalization matrix (TGM; face-watch: Figure 3D, other comparisons: Figures S5D-S5E). Successful decoding between time points (off-diagonal decoding) indicates that the direction in state space that discriminates between the categories remains stable in time. Thus, the rectangular temporal generalization pattern we reveal in occipitotemporal regions, and especially VT, indicates a highly stable, time-invariant category representation, as it shows that classifiers trained during the onset response were able to distinguish between the categories during the sustained response, and vice versa. This finding was also replicated at the single-patient level (Figures S5A and S5B). In contrast to this temporal invariance in occipitotemporal areas, category information in the PFC and Par did not generalize for the entire presentation of the stimulus (Figure 3D).

### Category information in visual, but not frontoparietal, regions tracks stimulus duration

**Figure 4.**
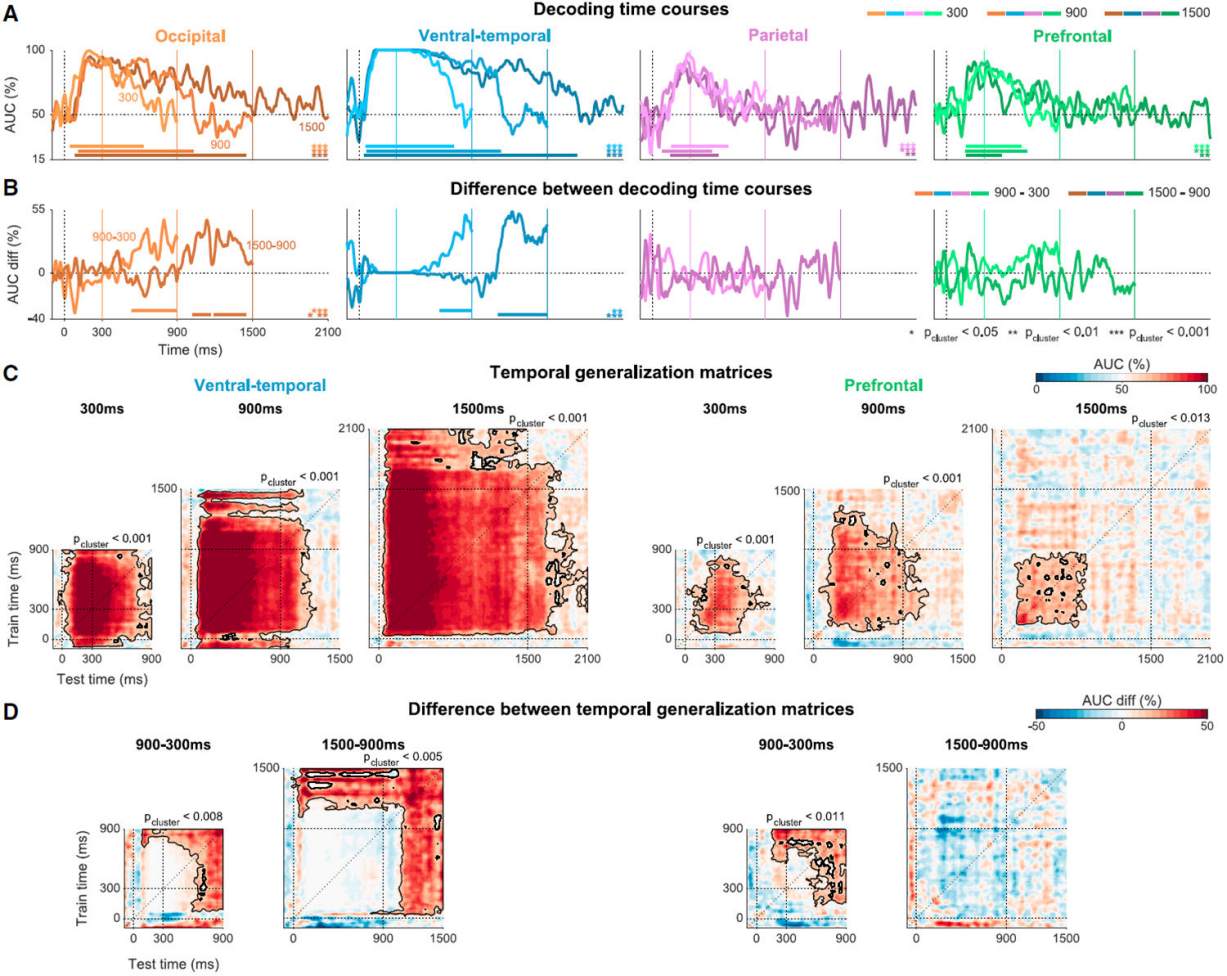
Category information in visual, but not frontoparietal, regions tracks stimulus duration (A) Decoding dynamics per duration (darker lines correspond to longer stimuli, offsets marked by corresponding vertical lines). Horizontal bars of corresponding color: significant decoding clusters (cluster permutation test); p values are indicated by bottom-right corner asterisks (corresponding to the cluster temporal order). Traces are cropped 600 ms after stimulus offset (shortest ISI). See also Figures S7A-7E. (B) Difference of decoding time courses (1,500–900 ms, dark lines; 900–300 ms, bright lines). Statistical testing and notations as in (A). (C) TGMs per duration (see Figure S7F for the other regions). Dashed lines: stimulus onset and offset and the diagonal (corresponding to the dynamics in A). Black contours: significant clusters; corresponding p values shown above each TGM. (D) Comparison between durations (notation and statistical testing as in C). (A–D) Occ and VT shown for patients S4-S10 (similar for S1-S3, Figure S7B). PFC is shown for S4-S10 as well (no responsive PFC electrodes for S1-S3). Par is shown for S1-S3 (not significant for S4-S10). See STAR Methods and Tables S1F and S1G for the rationale behind the split. All panels depict face-watch decoding (object-watch shown in Figure S7D).

The previous sections focused on responses to stimuli presented for 900 ms or longer. To ensure that the sustained decoding we found in visual sensory regions corresponds to the ongoing presence of the visual stimulus and does not merely reflect a prolonged onset response, we repeated the analysis separately for each duration (300, 900, and 1,500 ms). To allow a large enough number of images for each duration and category, the analysis was performed separately on patients S1-S3 and S4-S10 (see STAR Methods and Tables S1F-S1G for more details; Figure S7A shows electrode locations in each group). Category information in VT closely tracked the presence of the stimulus (with a short processing delay)—for all durations, significant AUC clusters emerged shortly after stimulus onset, persisted throughout stimulus presentation, and then subsided with a delay of approximately 450 ms for the 300 ms stimuli and 230 ms for the two longer stimuli (face-watch comparison; Figures 4A and S7B-S7C). Occ showed a similar pattern, with somewhat shorter offset delays. In both regions, the direct time-resolved contrasts between decoding images in different durations (900–300 and 1,500–900 ms) were statistically significant following the offset of the shorter stimulus (Figure 4B; all p_largest-cluster_ < 0.006). In contrast, decoding in the Par and PFC did not correspond to the duration of the stimulus (time course differences all p_cluster_ > 0.2). This conclusion is also supported by decoding of other category comparisons (Figure S7D) and by decoding using only positively responding electrodes (Figure S7E). This distinction between representational dynamics in sensory and frontoparietal regions was also evident by comparing the TGMs elicited by stimuli of different durations (VT and PFC, Figures 4C and 4D; Occ and Par, Figure S7F)—only occipitotemporal areas evinced time-invariant representations tracking the stimulus duration up to 1,500 ms, and this representation was stable in time.

To conclude, despite the drastic change in overall response amplitude (Figures 1 and 2), visual sensory areas maintain time-invariant category representations, tracking the duration of the stimulus. The PFC and Par cortex reveal reliable content representation but only transiently and independent of stimulus duration.

### Exemplar representation is sustained and stable in sensory regions and transient in frontoparietal regions

Perception is more than recognizing categories. For example, when looking at a face, we do not merely see a face, we see a specific instance of a specific face. We therefore turned to examine exemplar-level information using representational similarity analysis (RSA).^33,34^ RSA captures the representational structure by focusing on the pairwise dissimilarities between neural responses to each pair of images, grouped in a representational dissimilarity matrix (RDM). We quantify neural dissimilarities as 1-Pearson correlation, but, with few exceptions, which we note explicitly, similar results were obtained using Euclidean distance dissimilarity (Figures S8 and S9). We consider a region as representing exemplar information if the representational structure, conveyed by the dissimilarities, is reliable across separate repetitions of the same exact stimuli. Thus, we focus on 60 images viewed by five of the patients at least twice (for 900 ms or longer, as in previous analyses). Results from a larger group of eight patients (with only 18 shared images) are shown in Figures S8 and S9 (see STAR Methods for more details and Figure S8A for electrode locations in each subgroup).

**Figure 5.**
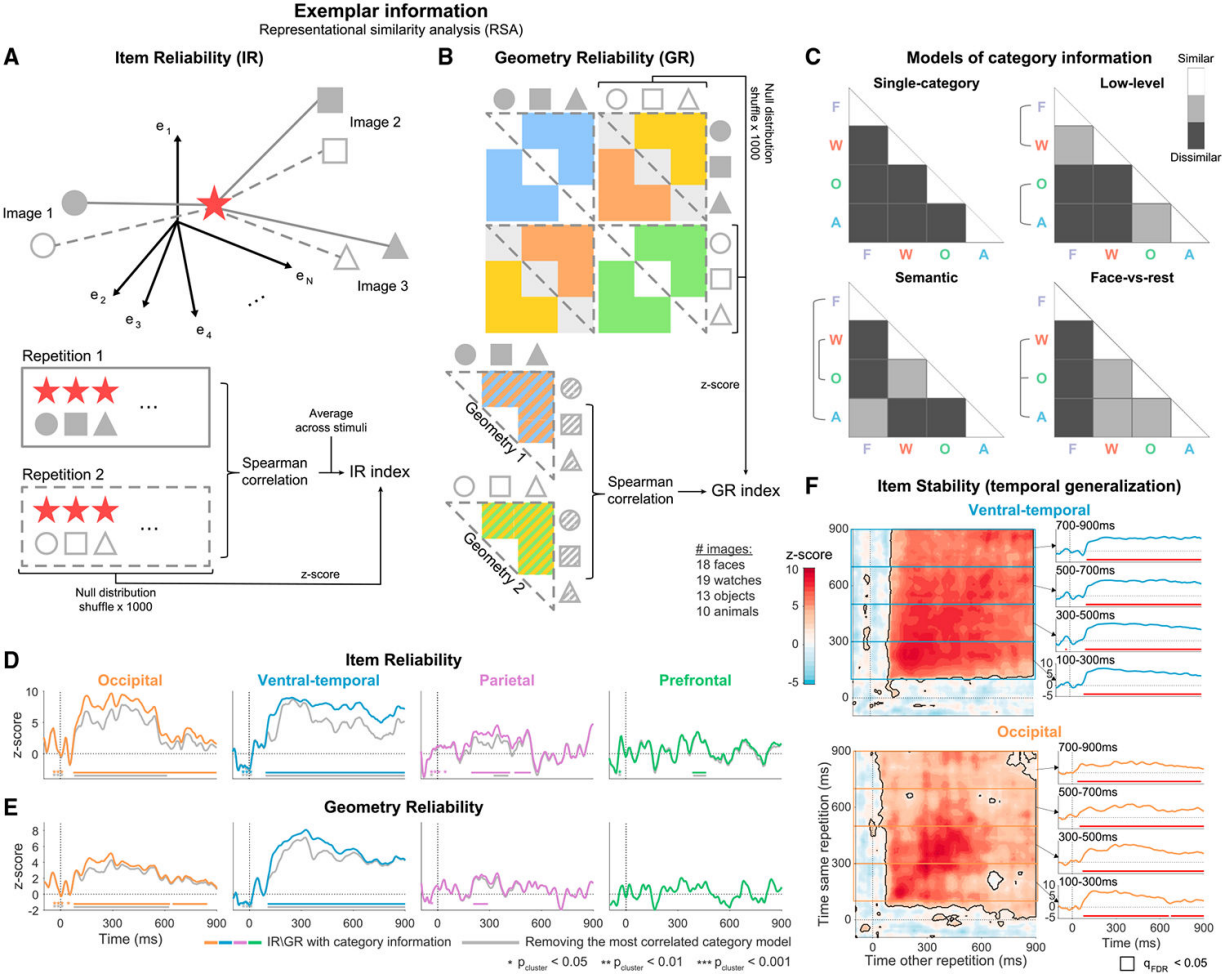
Exemplar information is sustained and stable in sensory regions and transient in frontoparietal regions (A) Schematic illustration of item reliability (IR): for each image (red star), we compare the vector of dissimilarities with all other images in repetition 1 (full shapes) with the vector of dissimilarities with all other images in repetition 2 (empty shapes). (B) Geometry reliability (GR): we first compute the dissimilarities between all images in both repetitions, resulting in a symmetric matrix with four distinct representational structures (top). Pairs of geometries are averaged to yield geometries 1 and 2, and the two geometries are correlated. See STAR Methods for more details about both reliability metrics. (C) Models of potential category information in the representational geometry. All models assume that exemplars within each category are similar to each other and dissimilar to other categories. Three of the models add a hierarchy of similarity between categories (STAR Methods). (D–E) IR and GR dynamics. Colored lines: full representational geometry (see Figure S8 for control analyses); gray lines: after partialing out the model explaining the most category information (see Figure S10 for more details about the calculation and removal of other category models; Figure S11 for single-category results). Horizontal bars of the same color mark significant clusters (cluster permutation test); p values indicated by bottom-left corner asterisks (corresponding to the cluster temporal order). Dashed lines: stimulus onset and chance level (no single-item information). (F) IR temporal stability (GR: Figure S9B; removing category information: Figures S10D and S10E; other regions: Figure S9A; other controls: Figures S9C-S9E). Notations as in Figure 3D. (D–F) Images presented at least twice with duration ≥900 ms.

To examine the reliability across repetitions, we designed two complementary metrics, both computed on a time point-by-time point basis. Item reliability (IR) captures the reliability of each stimulus representation within the overall geometry of responses by measuring separately the reliability of the response to each stimulus relative to other stimuli (Figure 5A). Geometry reliability (GR) captures global aspects of the representational structure by comparing the full dissimilarity structure across repetitions (Figure 5B; averaging different pairs of geometries or correlating each pair separately before averaging led to similar results). Note that both IR and GR compare dissimilarities between and within repetitions. Thus, by design, they capture not only preservation of the dissimilarity structure but also of state-space location (see STAR Methods for more details). Finally, both metrics are tested against surrogate distributions generated by shuffling stimulus identity (*Z* scoring shown in Figures 5A and 5B), and thus both metrics also reflect discriminability of single exemplars by the geometry (if multiple exemplars occupy similar positions within the geometry, shuffling them will not alter the geometry, and this will reduce the IR and GR scores).

Starting with VT, we found highly reliable exemplar representation sustained throughout stimulus presentation (IR and GR clusters extending to 900 ms, p_cluster_ < 0.001; Figures 5D and 5E, colored lines; see Figures S8B-S8C for additional regions not shown in the figure). We further tested for temporal invariance of the representation by comparing dissimilarities between time points (analogous to decoding temporal generalization) and found highly stable representation (high off-diagonal stability; IR: Figure 5F, GR: Figure S9B; see Figure S9A for additional regions). To verify that our reliability metrics reflect coding of single exemplars rather than relying solely on the category structure that we observed previously (Figures 3 and 4), we repeated the calculation of both metrics after removing category structure from the representational geometry. This was done by partialing out from the neural RDM four models of potential category information, designed in accordance with the literature and the observed state-space trajectories (Figure 5C; see STAR Methods for more details). The gray lines in Figures 5D and 5E depict IR and GR dynamics after partialing out the model that was most strongly correlated with the RDM of the region (Figure S10A), thereby providing the strictest measure of category information (using other models led to similar or higher reliability scores, Figure S10; see also Figure S11 for exemplar reliability within single categories supporting the same conclusions). Both IR and GR remained sustained and stable in VT throughout stimulus presentation after controlling for category information (Figures 5D and 5E gray lines; Figures S10B-S10E). Using only positively responding electrodes, Euclidean dissimilarities, or data from the eight-patient group led to similar results (Figures S8D-S8F and S9C-S9E).

In Occ, exemplar representation was largely sustained and stable using correlation dissimilarity (both p_cluster_ < 0.001), but this result was less robust: representation was not fully sustained nor stable after removing the categorical structure, nor with Euclidean dissimilarity or the larger patient group (Figures 5D-5E, S8D-S8F, and S9C-S9E). Exemplars were also reliably represented in the frontoparietal cortex, though this effect was short lived and noisier than in visual sensory regions (IR significant in both regions, all p_cluster_ < 0.015; GR significant only in Par, p_cluster_ < 0.043, albeit not after accounting for category structure). Thus, we conclude that representation of exemplars is sustained and stable (time invariant) in visual sensory regions, though it is robust only in VT, and there is transient and weaker exemplar representation in the PFC and Par.

## DISCUSSION

Delineating the neural correlates of conscious experience is one of the most coveted yet most challenging goals of cognitive neuroscience and perhaps science at large,^1^ leading to multiple competing hypotheses.^14,35^ In different guises, the quest for the NCC typically involves looking for an isomorphism between a specific experience and a neural signal by contrasting two states: having an experience (being consciously aware) of a stimulus and not having one.^31,36^ Usually, this requires unnatural manipulations of the stimuli, for example by masking or by major manipulations of attention.^37^ Under these liminal conditions, observers sometimes experience stimuli and sometimes not, and neural signals are then compared. These are powerful paradigms, especially for examining processing without conscious awareness, but in many cases, determining whether a stimulus was genuinely not experienced is difficult, and when experience is present, it is typically impoverished due to the manipulation.^38^

As our results show, manipulation of presentation duration of visible stimuli provides an illuminating alternative, as neural correlates of experience should account for the full temporal extent of experience, not only the experience of onsets or changes, which previous studies have focused on. We reveal that despite diminishing response magnitude (Figures 1 and 2), the pattern of activation across recording sites in Occ and especially the ventrotemporal cortex contains sustained and stable (invariant) information about the visual percept at both the category (Figure 3) and exemplar levels (Figure 5), corresponding to the duration of the stimulus (Figure 4). These properties are commensurate with the introspective intuition of ongoing, continuous perceptual experience. In contrast, we found a burst (“ignition”) of visual information in the PFC and Par cortex, even though no report was required. This representation lasted for a few hundred milliseconds after onset and did not correspond to the duration of the stimulus. This suggests that frontoparietal regions may be involved in updating perceptual experience, including when no overt response is needed, commensurate with a more discrete aspect of perceptual experience.^4,5^

### The temporal structure of experience

Introspectively, consciousness feels like a continuous flow of experiences, progressing in “real time” with events in the environment. However, this prevalent intuition has been challenged on both empirical and philosophical grounds.^3,39^ First, neural transmission takes time, and moreover, processing delays vary between modalities and even between different features in the same modality. Second, perception often requires integration of a temporal interval rather than a momentary instance (e.g., for perceiving motion or melodies). Perception of intervals is also suggested by postdictive effects, when a presented stimulus alters the way we perceive prior stimuli.^2,40^ One well known example is the “color phi phenomenon”^2^: two differently colored discs are flashed sequentially in different positions on the screen, but even though the stimuli do not change their position, the experience is of one disk moving between the two locations and changing color midway. Both the direction of movement and the color are fully unpredictable before the appearance of the second disk, meaning that the precept during the time interval between the flashes must be a retrospective “filling in.” One proposal to resolve this puzzle is postulating discrete perception^4,5^—if no perception at all occurred before the second flash, the full perceptual event can be organized unconsciously and experienced without any inconsistencies. However, the mechanism behind these types of effects is still studied, and continuous solutions have also been proposed.^3^

In the context of our paradigm, the introspective subjective percept is of stable continuous images with varying durations. Taken at face value, this would suggest that the sustained and stable visual representations in the ventral visual stream underpin our ongoing conscious experience. However, to the extent that perception is composed of discrete samples, each generating a transient ignition, the frontoparietal response would correspond more directly to experience. Thus, distinct representational dynamics, mapping to distinct hypotheses about the temporal nature of experience, seem to coexist in different regions. Rather than being mutually exclusive, these representations may be related to different aspects of a multifaceted experience or may interact hierarchically to form our ongoing conscious experience (see Singhal and Srinivasan^40^ for one such proposal). Altogether, the results show a deep connection between the classical NCC problem, which largely emphasized the anatomical underpinnings of consciousness, and the long-standing debate about the temporal nature of awareness. Finally, our results do not rule out a continuous role of the PFC in monitoring or supporting this representation in a manner that the does not represent the specific content of visual experience.^30^

### The role of PFC in perceptual awareness

Prefrontal involvement in perceptual awareness was questioned recently by several studies that reported minimal or no PFC activation when subjects are not required to report awareness overtly on a trial-by-trial basis^41,42^ (so called “no-report paradigms”^8^). These findings suggested that PFC is not involved in experience per se, but in reporting it (though see Dellert et al.^43^ for a recent no-report study in humans that did find PFC awareness effects and see Kapoor et al.^44^ and Bellet et al.^45^ for related findings in monkeys). Unlike the aforementioned no-report paradigms, the trials we analyzed, while not requiring a response, were task relevant, as the subjects had to decide whether the stimulus belonged to a target category. Nevertheless, our findings show human stimulus-specific representations that are not associated with an overt report and are not mapped to task distinctions.^46^ That is, we find in the PFC highly accurate decoding between image categories that are non-targets. Moreover, the task only required discrimination of category information, yet we also find reliable exemplar representation. Thus, contrary to recent claims that the PFC manifests only non-specific task-related activity,^47^ our results support content representation in the PFC following the onset of a new stimulus.

### Implications for specific theories of consciousness

An ongoing adversarial collaboration (COGITATE), aiming to adjudicate between two prominent theories of consciousness, IIT and GNWT, has adopted our multiduration paradigm as one of two key tests^14,48^ and provided detailed predictions by the two theories regarding this paradigm. IIT predicts that neural activity in a “posterior hot zone,” including Occ and VT regions, will persist with a stable representation as long as the visual experience persists, while it is largely agnostic about prefrontal involvement. GNWT predicts transient onset and offset representations without a sustained component in the PFC, including when no overt behavioral responses are performed. Both predictions bore out in our study—posterior (visual) areas showed stable persistent representation, tracking the duration of the stimulus, and the PFC (as well as the Par cortex) showed an onset (albeit no offset) ignition without persistent representation, absent behavioral responses. Thus, these predictions of IIT and GNWT are, in fact, not adversarial. Rather, the persistent representation in the occipitotemporal cortex and transient representation in the frontoparietal cortex may tap onto different components of experience.

### Distributed representation and the “experience subspace”

The notion that perceptual representations in humans are distributed, rather than local, has been suggested mainly based on fMRI findings^49,50^ and remains contested.^51^ The temporal stability of distributed representation reported here, in face of substantial local variability, supports the importance of distributed patterns. A shift from identifying the NCC with activation in specific neurons to the multivariate population response is also supported by a recent binocular rivalry study in monkeys showing that the same neurons in high-order visual cortex that represent the perceived stimulus also simultaneously code the suppressed image.^52^ Yet, a decoder trained on the population response was able to closely track the ongoing percept, indicating that even in the face of local heterogeneity, conscious content can be reliably coded at the population level.

The VT representations we identified were not only sustained but also highly stable. That is, the same classifier could discriminate categories from the onset response to the end of the presentation time, and similarly, the same representational geometry persisted throughout this time. Our results thus reveal an embedded subspace within the vast space of possible neural responses that maintains consistently the distinctions between visual categories and between exemplars within each category, despite considerable moment-by-moment variability in the full state-space response. The stability within this subspace also affords downstream regions with a stable readout of information about the visual percept.^53^ We propose that to the extent representations in sensory regions contribute to our conscious experience, it is the projection of the population response to this stable “experience subspace” that imbues each perceptual experience with its unique quality, while the variable activity in other dimensions may be used for other functions or may simply be the result of neural stochasticity. This may also explain why repeating stimuli seem identical despite the ubiquitous phenomenon of neuronal repetition suppression^54^—we suggest that as long as the response to a stimulus occupies the same location within the “experience subspace,” it will be similarly perceived regardless of the initial or sustained amplitude of response. Finally, it was recently proposed that the subjective quality of an experience is derived from the relation of its neural representation to other representations.^55,56^ The stable coding of the relational structure between neural responses to different exemplars that was found here is consistent with this view, under the premise that the subjective experience remains unchanged during the short presentation of the stimuli.

A related proposal has been put forward to explain the puzzling phenomenon of “representational drift,” that is, the observation that tuning properties of single neurons vary dramatically between trials or sessions, separated by seconds to many days, even in the face of consistent behavioral performance.^57,58^ This dissociation between stable behavior alongside variable representation resembles our findings of perceptual stability alongside variable neural response, albeit at a different temporal resolution. Several authors have suggested that representational drift is confined to a coding “null space,” that is, it influences population activity orthogonally to coding of task-related variables and therefore does not interfere with task performance^58-61^ (but see Rule et al.^62,63^). Most studies on representational drift involved single-unit recordings or local ensembles of neurons in laboratory animals, and our findings extend the notion to humans and more broadly distributed representations (see also a recent fMRI finding of representational drift across sessions^64^). Importantly, studies of representational drift typically treat representation as stable at the subsecond scale, yet our results show considerable response variability within a single event; therefore, more work is needed to connect the two phenomena.

### Conclusion

Which parts of the brain reflect our current perceptual experience and how their temporal dynamics correspond to the subjective experience are two major questions in the quest for understanding the neural correlates of conscious awareness. By manipulating stimulus duration, we were able to identify an important duality between these two aspects. In sensory regions, we find sustained and stable representation in an “experience subspace” embedded within the variable, diminishing neuronal responses. In frontoparietal regions, we found discrete (transient) content representation at stimulus onset. Thus, if the introspective subjective experience of a continuous stable percept given stationary input is accurate, it is suggested that the invariant sensory representation within the “experience subspace” imbues each perceptual experience with its unique, consistent quality. Yet, to the extent consciousness is discrete, this could be aligned with the discrete transient prefrontal representation. Thus, understanding the temporal resolution of experience will shed light on the anatomical location of the NCC, and delineating the NCC will in turn inform our understanding of the temporality of conscious experience.

### Limitations of the study

First, the PFC is a heterogeneous structure, with complex spatial organization^28,29^ and mixed selectivity at the single neuron level.^65^ Persistent information could be present in sites not sampled by our electrodes, which were placed solely based on clinical considerations and in this study were concentrated mostly on one hemisphere. The lack of sustained representation could also be related to the relatively small number of electrodes placed in the PFC, though reducing the number of electrodes in sensory regions to the same number available in the PFC did not substantially deteriorate the sustained representation observed in those regions. Additionally, the PFC may be involved in maintaining sustained continuous percepts using activity in low-frequency bands or in silent synaptic changes.^66^ For all these reasons, the absence of (sustained) activity should be taken with more caution than the presence of activity (as is always the case with null results). Second, the stimuli analyzed in this study were all above threshold, clearly visible images. Thus, it is possible that unseen, subthreshold images are similarly encoded over time. Future studies should test this possibility by comparing representation of seen and unseen sustained images and directly manipulate the duration of aware visual experience independent of the duration of the input, as in binocular rivalry.^52^ Finally, our use of the term “representation” is meant to denote neural patterns that correlate with external stimulus descriptors and should not be understood as implying mechanistic or functional roles,^67^ nor do we claim that the representation used by the brain is constrained by our ROIs, which were chosen to adhere to common divisions of the brain and to address arguments from the different consciousness theories.

## STAR★METHODS

### RESOURCE AVAILABILITY

#### Lead contact

Further information and requests for resources should be directed to and will be fulfilled by the lead contact, Gal Vishne (gal.vishne@mail.huji.ac.il).

#### Materials availability

The study did not generate new unique reagents.

#### Data and code availability

- De-identified human data have been deposited in a publicly available repository on OSF. DOIs are listed in the key resources table.
- All original code used for analyses and visualizations has been deposited in a public repository on Zenodo. DOIs are listed in the key resources table.
- Any additional information required to reanalyze the data reported in this paper is available from the lead contact upon request.

### EXPERIMENTAL MODEL AND SUBJECT DETAILS

Ten patients undergoing presurgical evaluation for treatment of intractable epilepsy (4 female, age (mean ± SEM): 41 ± 3.7, range: 19–65; for individual demographic details see Table S1A). Recordings were conducted in the Epilepsy Monitoring Unit (EMU). Seven patients were recorded in the Stanford School of Medicine, two in the California Pacific Medical Center (CPMC) and one in the University of California, San Francisco (UCSF) Medical Center. All patients gave informed consent approved by the University of California, Berkeley Committee on Human Research and corresponding IRBs at the clinical recording sites, in accordance with the Declaration of Helsinki. Results from the same dataset were previously reported in ref. 12.

### METHOD DETAILS

#### Stimuli and task

Patients viewed grayscale images, presented at the center of a uniform gray background, and extending approximately 5° of the visual field in each direction. Stimuli were presented on a laptop screen and responses captured on the laptop keyboard (Figure 1A). The images belonged to multiple semantic categories, including faces (~30%), man-made objects (watches: ~30%, other objects: ~18%) and animals (~10%). ~10% of images were targets (see below). The remaining images (<3%) were mostly houses or body-parts, which were not analyzed due to paucity of exemplars (see Table S1B for the number of stimuli viewed by each patient). Watches and other man-made objects were considered separately in this study since watch images, like the face images, were taken from a dataset of photos, while the other objects were illustrations. When comparing to face images, watches are considered a better control over low-level similarities, as the two categories share the overall round outline with internal details.^18^ To verify that information content tracked ongoing stimulus presentation, images were presented for variable durations (patients S1-S3: 300, 600, 900, 1,200 or 1,500 ms; patients S4-S10: 300, 900 or 1,500 ms). The probability of each exemplar appearing in each duration was uniformly distributed. A fixation cross was displayed between image presentations (inter-stimulus interval: 600, 750, 900, 1,050 or 1,200 ms). Image sequence was randomized for each patient, meaning that specific exemplars were viewed for different durations by different patients.

Patients were instructed to fixate at the center of the screen and respond with a button press to the appearance of rare targets (Figure 1B). In the main experimental condition, performed by all patients, the target was any image of a clothing item. The second experimental condition was performed by seven patients (S4-S10) in half of the blocks. In this condition patients were instructed to respond to two target types: (1) appearance of a clothing item (as in the main condition), (2) blurring of any image during the last 200 ms of presentation (see Gerber et al.^12^ for more details on the motivation of the dual task). Both target types together comprised ~10% of the trials and were not analyzed in this study, focusing on trials without report. Neural responses to non-targets did not differ between conditions,^12^ therefore data from both conditions was analyzed together.

#### Data acquisition and preprocessing

Patients were implanted with 64–128 subdural electrodes (total 1004), arranged in 1-dimensional strips and\or 2-dimensional grids (AdTech Medical Instrument Corporation). Electrodes were 2.3 mm in diameter, with 5 or 10 mm spacing between electrodes. Eight patients were implanted in the right hemisphere, and two in the left (Table S1C for individual electrode coverage). Two patients were additionally implanted with depth electrodes (total 28 electrodes), which were not analyzed in this study. We excluded an additional 35 channels which did not record any signal. Recordings were sampled at 1000 Hz (CPMC), 3051.76 Hz (Stanford, UCSF) or 1535.88 Hz (Stanford) and resampled to 1000 Hz offline. A high-pass filter was applied online to the signal at either 0.1 Hz (five patients, increased to 0.3 Hz for parts of the recording in two of the patients), 0.16 Hz (one patient) or 0.5 Hz (four patients). Electrodes manifesting ictal spikes or persistent noise were identified visually and removed from further analysis (0–38 electrodes per patient, total 125; only analyzed electrodes are plotted in visualizations of electrode positions). Electrodes were re-referenced offline to the average potential of all noise-free electrodes (per patient). Line noise (60 Hz and harmonics) was removed offline by a custom made notch filter, designed to remove persistent oscillations (not transients).^75^ All data processing and analysis was done in MATLAB (Mathworks, Natick, MA) using custom code or the toolboxes referenced in the key resources table. Visualization was done using custom code except violin plots which were created using ref. 74. Colormaps were created using colorbrewer (https://colorbrewer2.org/) with some adaptations.

#### Electrode localization

Electrodes were localized manually using BioImageSuite^70^ on a post-operative Computed Tomography (CT) scan co-registered to a pre-operative MR scan using the FSL software package.^71^ Individual patient brain images were skull-stripped and segmented using FreeSurfer.^72^ Localization errors (resulting from co-registration errors or anatomical mismatch between pre- and post-operative images) were reduced using a custom procedure which jointly minimizes the squared distance between all electrodes within a single electrode array or strip and the cortical pial surface. Individual patients’ brains and electrode coordinates were co-registered to a common brain template (FreeSurfer’s fsaverage template) using surface-based registration,^76^ which preserves the mapping of electrode locations to anatomical landmarks, and each cortical surface was resampled to a standardized mesh using SUMA^73^ (see Gerber et al.^12^ for more details). Cortical electrodes were assigned to one of six anatomical regions of interest (ROIs) based on the FreeSurfer automatic parcellation (ventral-temporal, occipital, prefrontal, parietal, sensorimotor and lateral-temporal; Figure 1C and Table S1D). Prefrontal electrodes were further divided into lateral-prefrontal and orbitofrontal cortices, and occipital electrodes were divided into retinotopic and non-retinotopic based on a probabilistic map of visual topographic regions^27^ (see electrode locations in Figure S1D). Twelve noise-free electrodes located over medial regions (mostly in the precuneus or cingulate cortex) were excluded from further analysis due to their paucity. Visualization of electrode positions was based on surface registration to an MNI152 standard-space T1-weighted average structural template image.

#### High-frequency activity estimation

We focus analysis on high-frequency activity (HFA, 70-150 Hz), previously shown to track firing rate in humans^19,20,77^ and other primates.^78,79^ We excluded the low-gamma range used in our previous study with this data, as it was shown to manifest distinct spectral and functional properties.^80,81^ To estimate the HFA time course we band-pass filtered the whole signal in eight 10 Hz sub-ranges between 70 and 150 Hz (EEGLAB’s FIR Hamming window, function ‘pop_eegfiltnew’^68^). We then extracted the instantaneous amplitude in each band using the Hilbert transform and normalized by dividing the signal by the mean amplitude in that range. Finally, we averaged the amplitude traces from all bands. Normalization was done to account for the 1/f profile of the power spectrum, which results in reduced contribution of the high frequencies relative to the lower frequencies. Trial segments were defined around each stimulus onset, and baseline corrected by subtracting the mean HFA signal in the 300 ms prior to stimulus onset from the entire trial segment. Trials containing excessive noise from −300 ms to 1,600 ms around each onset were excluded from analysis. The resulting HFA time courses were smoothed by a 50-ms moving window (smoothed time courses are used in all analyses unless noted otherwise).

### QUANTIFICATION AND STATISTICAL ANALYSIS

In the following sections we describe the four parts of the analysis in detail: Single electrode responses, Multivariate state-space responses, Decoding of category information, and Exemplar specific information. Following the description of the dependent measures in each section, we describe the approach to statistical analysis. Note that we use a multiverse approach, testing hypotheses in multiple ways to ensure the robustness of the results.^82^

#### Single electrode responses

##### Visual responsiveness

Responsiveness was tested separately for each of the four categories (considering watches and other objects separately), in four non-overlapping “stimulus-on” windows: 100-300 ms, 300-500 ms, 500-700 ms and 700-900 ms after stimulus onset, considering only trials with durations of 900 ms or longer. For each category and each time-window, we compared the mean HFA signal during the “stimulus-on” window to the mean HFA signal 200 ms prior to stimulus onset (*two-tailed* paired t test; averaging was done prior to smoothing the HFA trace to avoid information leakage between windows). We used Bonferroni correction across windows, and FDR correction (Benjamini-Hochberg procedure^83^) across electrodes, thus, electrodes with q_FDR_ < 0.05/4 in at least one of the four windows were considered responsive to that category. As the test was two-tailed, electrodes were considered responsive both when activity during the “stimulus-on” window increased relative to the pre-stimulus window, and when it decreased. Changes in response sign between windows or categories were rare (<5% of responsive electrodes), thus, electrodes were classified as positively responding (increase from baseline; 346/430 electrodes; Figures 1D-1E, and S1A-S1D) or negatively responding (decrease from baseline; Figure S1E) based on the sign of the sum of t-statistics from all tests (across all categories and all time-windows). Both types of electrodes were used in all analyses except where noted.

##### Response latencies and post onset attenuation

We first computed the mean HFA response of each electrode (using only stimuli from categories which the electrode was responsive to with stimuli duration of 900 ms or longer). Peak response magnitude (Figure S1A) and peak response time (Figure S1B) were defined as the maximal HFA value between 0 and 900 ms after onset and the time point when this was achieved, respectively. Relative attenuation of response magnitude was defined as the difference between the peak response and the mean response 800-900 ms after onset, scaled by the peak response (Figure 1E):

$$\text{response attenuation}\;(\%\;\text{from peak})\;:\;100(P_{resp}-E_{resp}){∕}_{P_{resp}}$$

where $P_{\text{resp}}$ is the peak response magnitude and $E_{\text{resp}}$ is the mean response 800-900 ms after onset. Comparison between regions was performed using one-way ANOVA followed by post-hoc tests using Tukey-Kramer method.

##### Category selectivity

Electrodes were defined as category selective if they showed differential responses to stimuli of the four categories in at least one of the four windows used above, for stimuli longer than 900 ms (one-way ANOVA, Bonferroni corrected across time-windows and FDR corrected across electrodes, similarly to the responsiveness criterion; Figure S1F). For each category selective electrode, we defined a selectivity time course by extracting the percent of variance in responses explained by category information on a point-by-point basis (100·*η*^2^, where *η*^2^ is the effect size measure from a point-by-point one-way ANOVA; Figures 1F-1G, and S1G-S1H).

##### Multivariate state-space responses

The main multivariate analyses (including decoding and exemplar analyses) were performed grouping together responses from multiple patients (c.f.^84,85^). Yet, to ensure no result is driven by a single patient we repeated the central analyses in single patients and found highly consistent results (Figures S5 and S6).

##### Analysis of state-space trajectories

The multivariate neural state at a specific time instance $\vec{s}{\;}^{t}=(s_{1}^{t},s_{2}^{t},\ldots ,s_{E}^{t})$ is defined as the pattern of recorded activity across electrodes^21-23^ ($s_{e}^{t}$ is the response of electrode e at time t and E is the number of responsive electrodes in a region). State-space trajectories record the changing multivariate states across time. To quantify the multivariate response magnitude (distance from baseline; Figures 2, S2, S3A and S3B) we computed the point-by-point Euclidean distance of the trajectory from the neural state prior to stimulus onset. Since all trials were baseline corrected, this is equivalent to computing the L^2^ norm of the vector of responses across electrodes. Attenuation was calculated similarly to the univariate case (Figure S2B).


$$\text{multivariate response (distance from baseline) at time t}\;:\;\|\vec{s}{\;}^{t}\|=\sqrt{\sum_{e\;=\;1}^{E}(s_{e}^{t}{)}^{2}}$$

State transition speed (Figures S2C and S2D) was quantified as the distance traveled by the neural state-space trajectory in 1 ms (corresponding to our sampling rate).


$$\text{state transition speed at time t}\;:\;\|\vec{s}{\;}^{t}-\vec{s}{\;}^{t-1}\|$$

To quantify whether the neural trajectories tracked the duration of the stimulus we computed the time point by time point distance between the responses to stimuli of different durations (Figures S3C and S3D).

$$\text{distance between trajectories at time t}\;:\;\|\vec{s}{\;}_{1}^{\;t}-\vec{s}{\;}_{2}^{\;\;t}\|$$

where $\vec{s}{\;}_{1}$ and $\vec{s}{\;}_{2}$ are response trajectories to different stimulus durations.

To evaluate category information in the multivariate trajectories (Figure S3E) we calculated the dispersion between response trajectories to different categories: First, we computed the square of the Euclidean distance (L^2^ norm squared) of each trajectory from the mean response across all categories. We defined the category selectivity index as the square root of the mean of these distances (analogous to computation of standard deviation in a one-dimensional distribution), and z-scored relative to a permutation null distribution as described in the next section.

$$\text{category selectivity index at time t}\;:\;\sqrt{\underset{C}{\text{mean}}\|\vec{s}{\;}_{C}^{{}^{t}}-\underset{C}{\text{mean}}\left(\vec{s}{\;}_{C}^{{}^{t}}\right){\|}^{2}}$$

where $\vec{s}{\;}_{C}^{{}^{t}}$ is the neural response to category C at time t, and $\underset{C}{\text{mean}}$ is the average over all four categories.

##### Statistical testing and confidence intervals

To evaluate statistically the dependence of multivariate response trajectories on stimulus duration (Figures 2B and S3A-S3D) we used permutation testing with max-statistic control for multiple comparisons^86^ (N_perm_ = 1,000, separate permutations for 900-300 and 1,500-900 comparisons). Since stimulus duration is expected to influence the response only at the times when one stimulus is still presented and the other is not, we considered only time points between 300 and 900 ms for the former comparison and between 900 and 1,500 ms for the latter (results were highly similar considering the entire time course). In each permutation we shuffled the duration labels across trials, before averaging the trials of each duration to construct the surrogate duration-specific state-space trajectory and computing the relevant difference statistic for each time point. Thus, we created N_perm_ permutation statistic time-series (N_perm_ x N_time_ matrix). Next, we computed the mean and standard deviation across permutations for each time point (column means and standard deviations) and used these to standardize (Z score) all permutation time-series. To create the surrogate distribution, we extracted from each standardized permutation time-series the maximal z-value across all time points. We also z-scored the values of the non-permuted (true label) statistic in the same manner, using the means and standard deviations of the permutation matrix (as under the null hypothesis the true statistic comes from the same distribution as the permutation statistics). Finally, time points when the unpermuted z-scored statistic was larger than 95% of the null distribution were considered significant (one-sided test). The procedure to test for significant category information (Figure S3E) was similar, except that we shuffled the category affiliation across trials and all time points were considered in the calculation.

Confidence intervals (Figures S2B and S2D) were computed using a jackknifing approach^87^ (N_iterations_ = 1000, out of $\prod_{p\;=\;1}^{10}n_{p}$ possible combinations per category, where $n_{p}$ is the number of trials of that category viewed by patient p). In each iteration, for each patient, the state-space response trajectory to each category was estimated by averaging the response to $n_{p}\;-\;1$ trials (separately for each region). We then merged the response trajectories of single patients (each with its own electrodes) to form a single response trajectory with all electrodes in each cortical ROI. Percent attenuation and speed difference for each iteration were computed in the same way as for the neural response calculated based on all images, and the distribution across iterations is provided as the confidence interval.

#### Decoding category information

##### Time point by time point classification

To quantify the representation of category information in each cortical region, we trained for each pair of categories a set of linear classifiers (one per time point) to distinguish between trials when one category was presented (e.g., faces) vs. the other category (e.g., watches), using the HFA amplitude of all responsive electrodes in the region as features^25^ (Figure 3A). For all analyses under this section (Figures 3-4 and S4-S7) the data was downsampled to 200 Hz to reduce computation time. To avoid overfitting, decoding analyses were carried out using five-fold cross validation, and to minimize variability stemming from the stochasticity in fold assignment, we repeated the five-fold cross validation procedure five times and averaged the results. In each iteration of the calculation, the category containing more trials was undersampled to balance the number of trials in both categories and we ensured that both categories were represented roughly equally in all folds.

We used regularized Linear Discriminant Analysis (LDA),^88^ as implemented in the MVPA-Light toolbox^69^ (downloaded on February 4th, 2021). LDA attempts to find a one-dimensional projection of the data (a linear weighting of electrodes) with maximal separation between the categories. This is done by simultaneously trying to maximize the distance between the mean responses to each category (“signal”), while minimizing the variability in responses to each of the categories (“noise”). Formally, classifier weights (the projection vector) are computed according to:

$$\text{LDA classifier weights}\;:\;\vec{w}=\Sigma^{-1}\left(\vec{\mu }{\;}_{{}_{1}}-\vec{\mu }{\;}_{{}_{2}}\right)$$

where $\vec{\mu }{\;}_{1}$ and $\vec{\mu }{\;}_{2}$ are the mean responses to category 1 and category 2 (e.g., faces and watches), and Σ is the pooled covariance matrix (summing covariances of each category, weighted according to the number of exemplars per category). The predicted category for each trial at each time point is given by comparing $\vec{w}{\;}^{{}^{T}}\vec{s}{\;}^{{}^{t}}$ to a set threshold θ:

classifier prediction:{w→Ts→t>θ→category 1w→Ts→t<θ→category 2}

The threshold can be modified to alter the balance of true positives (trials from category 1 classified as category 1) and false positives (trials from category 2 wrongly classified as category 1), resulting in the receiver operating characteristic curve (ROC), which depicts the false positive rate (FPR) and the true positive rate (TPR) for each threshold θ. We quantified the classifier’s performance by using the area under the ROC curve (AUC):

$$\text{area under the curve (AUC)}\;:\;\int_{0}^{1}\text{TPR}({\text{FPR}}^{-1}(x))dx$$

When the classifier contains no category information the AUC is equal to 0.5 (50%, chance level performance), and when the classes are fully separable the AUC is equal to 1 (100%, perfect performance). We used AUC, as opposed to accuracy, since AUC is more robust to unequal class sizes, and is not influenced by changes in the overall magnitude of the response when the pattern across electrodes remains similar.

To reduce the influence of random noise fluctuations on the weight estimate we used a shrinkage estimator for the covariance matrix. Instead of using empirical covariance in the equation above, we use a combination of the empirical covariance (Σ) and the identity matrix (I), weighted according to the regularization parameter λ:

$$\text{shrinkage regularization of the covariance matrix}\;:\;\hat{\Sigma }=(1-\lambda )\Sigma +\lambda \frac{trace(\Sigma )}{p}I$$

where p is the number of features of the classifier. Multiplying the identity matrix by the mean value on the diagonal ensures that the trace of the covariance is preserved, which helps to mitigate the bias introduced by this step. The regularization parameter l was estimated using the Ledoit-Wolf formula^89^ (default implementation in MVPA-Light,^69^ for more explanation on the rationale of shrinkage regularization see Blankertz et al.^90^).

The main decoding analyses were carried out on responses to 92 unique images which were seen by all patients for ≥900 ms (faces, 30; watches, 32; other objects, 18; animals, 12; Figures 3B-3D, S4, S5C-S5E, and S6C). To avoid overfitting,^25^ repetitions of the same image were averaged to ensure it is not used simultaneously in the training and testing sets (number of repetitions mean ± std across patients and unique exemplars: 1.5 ± 0.6, range 1–4, similar for all categories). Using only the first repetition of each image instead of averaging the repetitions elicited highly similar results. Analyses performed on data from single patients used all unique exemplars viewed by each patient for ≥900 ms (mean ± SEM unique exemplars across patients: faces, 62.5 ± 4.2; watches, 65.5 ± 4.5; objects (non-watch), 39 ± 2.3; animals, 22 ± 1.1; Figures S5A, S5B, S6A, S6B and S6E). Comparison of bipolar and average montages was performed on patients S8 and S10 together (faces, 54; watches, 58; other objects, 34; animals, 20; Figure S6D). To run analyses for each stimulus duration separately (Figures 4 and S7) we split the patients into two groups (patients S1-S3, patients S4-S10), in order to increase the number of exemplars seen in the same duration by all patients in the group (due to the randomization of the image sequence the full group shared on average only 4.8 exemplars per category and duration). The number of exemplars in each category and duration shared by the full patient group and each subgroup are shown in Table S1F.

##### Control for potential ocular muscle artifacts

Previous studies have shown that HFA activity in the proximity of the orbit may be confounded with saccade related activity from extraocular muscles (spike potentials).^91,92^ While these studies explicitly showed no contamination of responses in OFC (as opposed to the temporal pole),^91^ and patients were instructed to fixate during our task, we nevertheless performed two control analyses to ensure that the ability to decode category information from OFC was not driven by eye-muscle activity.

###### Decoding using bipolar reference montages (Figure S6D):

We re-referenced the data after extraction of HFA traces by subtracting from each electrode the mean HFA activity in all adjacent electrodes on the same grid. Both responsive and non-responsive electrodes were included for re-referencing but only contacts centered on responsive electrodes were used for classification. Two patients had enough electrodes placed over OFC (inset above Figure S6D): S8: 16 OFC electrodes, 5 excluded (4 placed on arrays which were mostly over the temporal pole; 1 containing epileptic activity), resulting in 11 electrodes (5 responsive); S10: 10 OFC electrodes (6 responsive) + 2 LPFC electrodes on the same grid (used for re-referencing).

###### Decoding in time-windows without any saccades (Figure S6E):

Eye-tracking was not possible in the EMU setting, yet we were able to reliably identify the timing of saccades in one patient (S8), by detecting the saccadic spike potentials in an electrode placed over the temporal pole, behind the right eye (same electrode used in^12^). Following ref. 75, we first convolved a template for the saccadic spike potential with the signal from the relevant electrode (for the template we used the validated publicly available “matched filter”^75^). Second, we marked all points higher than 3 standard deviations of the entire convolution time course. Finally, we denoted the onset of each marked time-range as a saccade. This produced the expected saccade modulation curve around stimulus onset (suppression followed by rebound; similar to Figure 8 in Gerber et al.^12^), supporting the assertion that the detected time points correspond to saccadic events. It was not possible to exclude all trials where a spike potential was detected between 0 and 900 ms, as this would exclude 90% of the relevant trials and leave just 1–2 unique images in the animal and (non-watch) object categories. To overcome this problem, we focused on four non-overlapping time-windows between 100 ms and 900 ms after stimulus onset and analyzed each window separately while excluding only trials where a saccade occurred within that time-window. Using this approach, we were able to use 67–82% of the images across time-windows (72–78% of images from each category). Decoding analysis was carried out on the mean activity in each time-window (unsmoothed data), resulting in one AUC value for each time-window and category comparison. We used only responsive OFC electrodes which were not adjacent to temporal pole electrodes.

##### Temporal generalization analysis

To assess whether coding of category information was stable in time we used the temporal generalization method^32^ (Figures 3D, 4C, 4D, S5 and S7F). In this method linear classifiers are trained on data from each time point separately, but tested on data from all time points, not only the one used for training. The result is a matrix of decoding values (Temporal Generalization Matrix, TGM), with the y axis indicating the training time point and the x axis indicating the testing time point. Successful decoding between time points indicates that the direction in state-space which discriminates between the categories remains stable in time (the specific cutoff between categories may change as we used AUC to evaluate classifier success, which does not rely on one fixed threshold).

##### Statistical testing

All statistical testing was based on permutation tests (N_perm_ = 1000; one-sided). To construct the null permutation distribution, in each permutation we permuted the category labels across trials and trained classifiers for all time points based on the permuted labels (we used the same set of permuted labels to preserve the temporal structure of the data). Peak decoding performance (Figure 3B) was statistically tested by comparing to the distribution of peak AUCs of each permutation (maximum across time points), equivalent to a max-statistic approach to control for multiple comparisons.^86^ Controlling for multiple comparisons for decoding time courses and TGMs was done using cluster-based permutations^26^ (details below; Figures 3C and 4, S4A, S4B, S5A-S5C, S6A-S6D and S7B, S7D-S7F). Cluster-based permutations are sensitive, yet they are insufficient to establish the precise latency or temporal extent of effects,^93^ therefore, we additionally employed point-by-point comparisons controlling the FDR^83^ whenever inferences about specific time points were required (q_FDR_ < 0.05; Figures 3C, 3D, S5 and S7C).

Cluster-permutation details: We selected all time points with AUC greater or equal to 60% (first-level threshold; other thresholds led to similar results), clustered the samples based on temporal adjacency (including both train and test times for TGMs) and extracted the sum of AUC values in each cluster (cluster statistic). To construct the null distribution, we performed the same procedure on each of the permutations, but only the maximal cluster value was retained in the null distribution. Clusters from the original (non-permuted) calculation that exceeded 95% of this distribution were considered significant (one-sided test).

Statistical comparison of decoding between regions (Figure S4B) and comparison of decoding of stimuli in different durations (Figure 4 and S7) was performed in a similar way, except the cluster first-level threshold was set to AUC difference of 10% between regions or duration conditions. For comparison between regions we used a two-sided test. In both cases we permuted the category labels and trained classifiers for each region (or duration) separately and formed the null distribution by subtracting the decoding results for different regions, or, in the duration case by subtracting the short duration (300 or 900 ms) from the long duration (900 or 1,500 ms) and extracting the max cluster statistic as described above. When we compared the decoding time courses between durations we considered only time points after the offset of the short stimulus, as we predicted a difference only when one stimulus was presented, and the other was not (results were nearly identical without this constraint). For TGMs we considered the full matrix as we were also interested in generalization between the onset and the time period after the offset of the short stimulus.

#### Exemplar specific information

##### Quantifying exemplar representation

To quantify exemplar-level information we used representational similarity analysis (RSA),^33,34^ which describes neural representation in terms of the relation between neural responses, that is, the geometry that the responses define. The representational geometry is fully captured by the set of all pairwise dissimilarities between the pattern of responses across electrodes to each pair of stimuli, grouped together in a representational dissimilarity matrix (RDM). We employed two dissimilarity measures – correlation dissimilarity (Figures 5, S8B-S8D, S8F, S9A-S9C, S9E, and S10-S11) and Euclidean dissimilarity (Figures S8E, S9D), as these are sensitive to different aspects of the response^94^:

$$\text{correlation dissimilarity}\;:1-r\left(\vec{s}{\;}_{A},\vec{s}{\;}_{B}\right)\;\;\text{Euclidean dissimilarity}\;:\|\vec{s}{\;}_{A}-\vec{s}{\;}_{B}\|$$

where $\vec{s}{\;}_{A}$ and $\vec{s}{\;}_{B}$ are the vector of HFA responses to stimulus A and B across electrodes (in a specific time point), $r(\vec{u},\vec{v})$ is the pearson correlation of $\vec{u}$ and $\vec{v}$ and ‖⋅‖ norm.

To avoid overfitting to incidental noise fluctuations, exemplar-level analyses were only conducted on stimuli which were viewed at least twice (akin to a cross-validation procedure), for 900 ms or longer. Four patients viewed only 27 exemplars twice from the analyzed categories, and one patient did not view any exemplar twice, thus, to ensure sufficient sampling of all categories we performed the main analyses in this section on a group of five patients who all viewed the same set of 60 unique images at least twice (faces, 18; watches, 19; other objects, 13; animals, 10; Figures 5, S8B-S8E, S9A-S9D, and S10-S11). Results from a larger eight patient group (excluding the patient who did not view any exemplars twice and one patient which had excessive noise in many trials) with only 18 unique exemplars (faces, 6; watches, 7; other objects, 2; animals, 3) are presented as a control in Figures S8F and S9E. Electrode locations of both groups are shown in Figure S8A.

We computed the dissimilarity structure separately for each repetition and time point and compared between repetitions (reliability analysis) and between time points (stability analysis) using Spearman correlation, which is more robust to changes in the overall magnitude of activity relative to Pearson correlation. As with the decoding analysis, for all analyses under this section (Figures 5 and S8-S11) the data was downsampled to 200 Hz to reduce computation time.

#### Representation reliability across repetitions

##### Item Reliability (IR; Figures 5D, 5F, S8B-S8F, S9A, S9C-S9E, S10B, S10D, and S11):

If exemplars are represented reliably across repetitions, the location of each exemplar within the neural geometry should remain consistent across repetitions. Thus, for each stimulus presentation (Figure 5A, red star), we computed the correlation of the vector of dissimilarities to all other images in repetition 1 (filled shapes) and the vector of dissimilarities to all other images of repetition 2 (empty shapes). We then averaged the obtained correlations across all images and repetitions, and z-scored the result using a permutation null distribution (N_perm_ = 1000). To construct the null distribution, for each image presentation we shuffled the stimulus identity of all images from the *other* repetition and repeated the process (i.e., if the presented image was from repetition 1 (as in Figure 5A) we shuffled repetition 2 and if the presented image was from repetition 2, we shuffled repetition 1).

##### Geometry Reliability (GR; Figures 5E, S8B-S8F, S9, S10C, S10E):

Our second measure of reliability captures global aspects of the representation by comparing the full dissimilarity structure across repetitions. We first computed the RDM between all stimulus presentations from both repetitions (Figure 5B, top), containing four distinct sets of dissimilarities: within repetition 1 (blue), within repetition 2 (green) and two between repetition 1 and repetition 2 (yellow and red). Second, we paired each within repetition dissimilarity set with one between repetitions dissimilarity set and averaged the dissimilarities in each pair (resulting in Geometry 1 and Geometry 2 in Figure 5B bottom; averaging different pairs of geometries or correlating each pair separately and then averaging led to similar results). Considering both within repetition and between repetition dissimilarities ensures not only preservation of the geometry across repetitions, but also a similar state-space location between repetitions. Third, we computed the correlation between Geometry 1 and Geometry 2 (unfolded into vectors). Finally, we z-scored the result using a permutation null distribution (N_perm_ = 1000), constructed by shuffling the identity of all *single exemplars* in repetition 2 and repeating the procedure. Importantly, in cases where some exemplars are represented similarly within the geometry (indistinguishable representations) this approach is likely to result in insignificant GR even if the geometry is fully maintained between repetitions. Thus, it is a test of both representational reliability between repetitions and of discriminability between exemplars.

###### Representation stability in time

We tested the temporal stability of the representation by comparing representational structures across time points (Figures 5F, S9, S10D, S10E, and S11C). This was done similarly to the reliability analyses, only representational structures were computed in different time points. IR stability for time points (t_1_, t_2_): for each stimulus presentation S, we first computed the dissimilarities between S at time t_1_ and all other exemplars in the *same* repetition as S also at t_1_ (y axis of stability plots), and then correlated this dissimilarity vector to the dissimilarities between S at time t_2_ and all *other* exemplars in the other repetition at t_2_ (x axis of stability plots). We then averaged the correlation across stimuli as in the standard IR calculation. GR stability for time points (t_1_, t_2_): we correlated Geometry 1 in t_1_ (y axis) with Geometry 2 in t_2_ (x axis). When t_1_ = t_2_ (diagonal of stability matrices) this is equivalent to calculation of IR and GR, respectively. Note that by correlating dissimilarity structures at different repetitions (for at least one of the exemplars), we avoid overfitting to spontaneous fluctuations unrelated to stimulus processing, which are known to exhibit many temporal dependencies.^95-97^

###### Accounting for category structure

To test whether exemplar-level information is present beyond category differences we designed four models of category information (Figure 5C) and tested whether exemplar-level information is reliable and stable after removing category information from the neural RDM using partial correlation (i.e., partialling out the model RDMs; Figures 5D, 5E, and S10). For IR we used a single row of the model RDM for each stimulus (treating the RDM as a symmetric matrix, but excluding the diagonal), and for GR we partialled out the full model RDM unfolded into a vector. All models assume exemplars within a category are similar to each other, and dissimilar to exemplars in other categories. In models 2–4 we add a hierarchy of similarities between categories (supported by previous studies^50,98,99^), such that categories belonging to the same higher order category are 50% similar. The models are (Figure 5C): (1) “Single-category” – no relation between categories, only a primary category distinction. (2) “Low-level” relation between categories based on low-level visual similarity – faces and watches form one high-order category (both are photos of round objects), and other (non-watch) objects and animals form the second (pointy illustrations). (3) “Semantic” (based on animacy) – faces and animals form one high-order category (animate) and all objects (watches and non-watches) form the second (inanimate). (4) “Face-vs-rest” – faces are distinct from all other categories, which are all grouped into one high-order category; we constructed this model due to the unique social importance of faces and the known specialization in face representation (also supported by our results, Figure 2A).

###### Statistical testing

All statistical testing was permutation-based (N_perm_ = 1000). Construction of the null distribution is detailed above (section “Representation reliability across repetitions”, different procedures for IR and GR). In each permutation, we used the same set of permuted labels for all time points to preserve temporal properties of the data. Z-scoring was performed in each time point separately, for both the original (non-permuted) score and the permutation results. Statistical testing and control for multiple comparisons was done similarly to the decoding analyses (all one-sided): Time courses were tested using cluster-based permutations^26^ (sum of reliability indices as the cluster-statistic). First-level threshold was set to z = 1.5 SD for the main analyses (Figures 5D-5E, S8 and S10B-S10C) and z = 0.75 SD for the single category analyses (adjusted to accommodate the larger temporal smoothing window in these analyses (100 ms vs. 50 ms in all other analyses); Figures S11B-S11D), in both cases similar results were obtained with other thresholds. The time courses of correlation between each category RDM model and the neural RDM (Figure S10A) were also corrected using cluster-based permutations, with the sum of the correlations as the cluster-statistic and ρ = 0.0391 as the first-level threshold (corresponding to a p value of 0.05). Stability (generalization) matrices were tested using point-by-point comparisons, controlling FDR^83^ (q_FDR_ < 0.05; Figures 5F, S9, S10D and S10E).

## Supplementary Material

1

2

## Figures and Tables

**Table:** KEY RESOURCES TABLE

REAGENT or RESOURCE	SOURCE	IDENTIFIER
Deposited data		
De-identified patient data	This study	https://doi.org/10.17605/OSF.IO/4HXPW
Software and algorithms		
MATLAB 2021a	Mathworks	https://www.mathworks.com/; RRID: SCR_001622
Custom code	This study	https://doi.org/10.5281/zenodo.8051237
iEEG_decoding_minitoolbox	This study	https://doi.org/10.5281/zenodo.8051195
Gals_RSA_toolbox	This study	https://doi.org/10.5281/zenodo.8049315
Time_resolved_stats	This study	https://doi.org/10.5281/zenodo.8049317
State_space_plot	This study	https://doi.org/10.5281/zenodo.8049319
EEGLAB toolbox	Delorme and Makeig^68^	https://sccn.ucsd.edu/eeglab/index.php; RRID: SCR_007292
MVPA-Light toolbox	Treder^69^	https://github.com/treder/MVPA-Light; RRID:SCR_022173
BioImageSuite	Joshi et al.^70^	www.bioimagesuite.org; RRID:SCR_002986
FSL software package	Jenkinson et al.^71^	https://fsl.fmrib.ox.ac.uk/fsl/fslwiki/; RRID:SCR_002823
FreeSurfer	Fischl^72^	https://surfer.nmr.mgh.harvard.edu/; RRID:SCR_001847
SUMA - AFNI Surface Mapper	Argall et al.^73^	https://afni.nimh.nih.gov/Suma/; RRID: SCR_005927
Colorbrewer	Cynthia Brewer and Mark Harrower	https://colorbrewer2.org/
Violinplot-Matlab	Bechtold^74^	https://doi.org/10.5281/zenodo.4559847

## Highlights

- ECoG reveals encoding of categories and exemplars for images of varying durations
- Sustained visual information is represented with varying dynamics across the brain
- Occipitotemporal distributed representations are sustained, tracking image duration
- Frontoparietal distributed representations are transient, following stimulus onset
